# Detection of botanical adulterants in saffron powder

**DOI:** 10.1007/s00216-023-04853-x

**Published:** 2023-08-17

**Authors:** Jana Ryparova Kvirencova, Klara Navratilova, Vojtech Hrbek, Jana Hajslova

**Affiliations:** Department of Food Analysis and Nutrition, University of Chemistry and Technology Prague (UCT Prague), Technicka 3, 166 28, Prague 8, Czech Republic

**Keywords:** Saffron, *Crocus sativus* L., Authenticity, Botanical adulterants, Metabolomic fingerprinting, UHPLC-HRMS/MS

## Abstract

Saffron is a unique spice obtained by drying stigmas of saffron flowers (*Crocus sativus* L.). Due to its high price, economically motivated adulteration occurs relatively often. The presented study aimed to develop an effective strategy for the detection of the following potential botanical adulterants used for a saffron substitution or dilution: safflower (*Carthamus tinctorius* L.), calendula (*Calendula officinalis* L.)*,* turmeric (*Curcuma longa* L.), achiote (*Bixa orellana* L.), red pepper (*Capsicum* spp.), mountain arnica (*Arnica montana* L.), beet (*Beta vulgaris* L.), and pomegranate (*Punica granatum* L.). A non-target screening strategy based on ultra-high performance reverse-phase liquid chromatography coupled to tandem high-resolution mass spectrometry (UHPLC-HRMS/MS) was employed for the analysis of an aqueous ethanol plant extract. By using multivariate statistical methods, principal components analysis (PCA), and partial least squares discriminant analysis (PLS-DA), for processing the generated “chemical fingerprints,” metabolites unique to the investigated plants could be identified. To enable routine saffron authenticity control by target screening, an internal spectral database was developed; currently, it involves 82 unique markers. In this way, the detection addition as low as 1% (*w/w*) of all analyzed botanical adulterants in admixture with saffron was possible. The developed method was used to control 7 saffron powder samples from the Czech market, and none of the monitored adulterants were confirmed.

**Supplementary information:**

The online version contains supplementary material available at 10.1007/s00216-023-04853-x.

## Introduction

Saffron has always been one of the world’s most precious spices. It is obtained by drying the stigma of *Crocus sativus* L., which belongs to the *Iridaceae* family [1, 2]. This plant is mainly grown in countries with warm and dry summers and cold winters, i.e., in Iran, Spain, or Greece. Stigmas from flowers are harvested by hand, without mechanization, which significantly increases the value of saffron spice [1]. In addition, approximately 150,000 plants are needed to produce 1 kg of this spice, which is also why saffron is known as the most expensive spice in the world [3, 4].

For food flavoring, saffron is sold in various forms. Stigmas are of the highest quality, but ground stigmas (saffron powder) are often sold for easier use. Existing data show that this form is prone to adulteration [5]. For instance, up to 33% of saffron samples are reportedly adulterated in the Chinese market [6]. The most common way is undeclared replacement or substitution of saffron using other plant material or its extract. Specifically, it may be undeclared additions of colored stamens [4, 7–10], styles [10–12], or petals [9, 10] of the saffron itself. For this purpose, petals of safflower (*Carthamus tinctorius* L.) [1, 2, 4, 6–8, 10–23], petals of calendula (*Calendula officinalis* L.) [1, 2, 4, 7, 10–13, 15, 17, 20], rhizomes of turmeric (*Curcuma longa* L.) [1, 2, 4, 7, 8, 10, 12, 15, 17, 18, 20, 21, 23], extracts of gardenia fruits (*Gardenia jasminoides* J. Ellis) [1, 4, 5, 7, 8, 17, 24–27], petals of mountain arnica (*Arnica montana* L.) [1, 2, 15, 17], dye from seeds of achiote (*Bixa orellana* L.) [2, 15, 17], petals of chrysanthemum (C*hrysanthemum morifolium* L.) [6, 16, 17], petals of hemerocallis (*Hemerocallis* sp.) [2, 15, 17], stigmas of crocus vernus (*Crocus vernus* (L.) Hill) [2, 15], dye from pomegranate fruits or petals (*Punica granatum* L.) [10, 17], dye from flowers of buddleia (*Buddleja officinalis* Maxim) [4, 7], dye from beet (*Beta vulgaris* L.) [10, 17], and another else also can be used.

The quality and classification of saffron are defined by the ISO 3632-1:2011 and ISO 3632-2:2010 standards, where the criteria for determining taste, color, aroma, moisture, minerals, foreign substances, and exogenous dyes are listed [28, 29]. The ISO standard recommends using spectrophotometry for verifying the authenticity of the saffron sample. The water–ethanol extracts are measured at three different wavelengths: 257 nm for picrocrocin, 330 nm for safranal, and 440 nm for crocin [29]. Using this method, it is possible to detect the lowest additions of safflower, calendula, and turmeric in the range of 10 to 20% of the total weight [30].

In addition to spectrophotometry, methods based on the combination of liquid chromatography (LC) and mass spectrometry (MS) [5, 9, 13, 16, 24] optionally supplemented with a diode array detector (DAD) or photodiode array (PDA) [26, 27, 30]. Furthermore, proton nuclear magnetic resonance (^1^H NMR) [3, 8, 14, 18] or molecular genetic methods [2, 4, 6, 15, 19] and others are often used. An overview of the selected publications is in Table 1.

**Table 1:** The overview of publications dealing with the detection of saffron adulteration with the addition of another botanical species

Method	Adulterants	The lowest detected addition of adulterants (% *w/w*)	Reference
UHPLC-HRMS/MS	Calendula Safflower	Not reported	[13]
UHPLC-HRMS	Petals of saffron Stamens of saffron	5	[9]
HPLC-HRMS	Gardenia	10	[5]
HPLC-HRMS	Gardenia	0.2	[24]
HPLC-MS/MS	Safflower Chrysanthemum Corn stigma	10	[16]
HPLC-PDA-MS	Calendula Safflower Turmeric	5 2 2	[30]
UHPLC-DAD-MS	Gardenia	15	[27]
LC-DAD-MS	Gardenia	Not reported	[26]
MALDI-MS/MS	Gardenia	Not reported	[25]
DRIFTS (diffuse reflectance infrared Fourier transform spectroscopy)	Stamens of saffron Calendula Safflower Turmeric Buddleia Gardenia	5 (based on mathematical and statistical calculations: 1–3)	[7]
DART-MS	Safflower Turmeric	20	[21]
GC-C-IRMS (gas chromatography–combustion–isotopic ratio mass spectrometry)	Calendula Safflower Rubia Style of saffron	5	[11]
^1^H NMR	Safflower	Not reported	[3]
^1^H NMR	Safflower	30	[14]
^1^H NMR	Stamens of saffron Safflower Turmeric Gardenia	20	[8]
^1^H NMR + ^1^H NOESY (nuclear overhauser effect spectroscopy) NMR	Safflower Turmeric	5	[18]
PCR (polymerase chain reaction)	Buddleia Gardenia Turmeric Safflower Calendula	Not reported	[4]
SCARs (sequence-characterized amplified regions)	Arnica Achiote Calendula Safflower *Crocus vernus* Turmeric Hemerocallis	1	[15]
DNA barcoding technique	Safflower Lotus flower Chrysanthemum Maize	Not reported	[6]
Specific and real-time PCR	Safflower	0.1	[19]
LIBS (laser-induced breakdown spectroscopy)	Safflower Calendula Turmeric	5 (based on mathematical and statistical calculations: 1.86)	[20]
UV-Vis spectrophotometry	Calendula Safflower Turmeric	10–20	[30]
NIR (near-infrared spectroscopy)	Stamen of lotus Stigma of corn	Not reported	[22]
Vis-NIR-HSI (visible-near-infrared hyperspectral imaging)	Safflower Style of saffron Calendula Rubia Turmeric	5	[12]
TLC (thin-layer chromatography) coupled with Raman spectroscopy	Safflower Turmeric	Based on mathematical and statistical calculations: 31.01 41.98	[23]

As shown in Table 1, only a few studies focused on more than four different potential adulterants. It is needed to mention that also the use of molecular genetic methods is very promising in terms of detecting the lowest possible addition of adulterants. However, in the case of using plant extracts for adulteration, DNA damage may occur; thus, the used adulterant may not be detected by this method [7]; also drying can cause DNA degradation.

In the presented study, liquid chromatography coupled with mass spectrometry technique as a tool for saffron adulteration was selected, which is nowadays commonly used in food/natural product composition studies. The ambition of this research was not only to involve a broad range of botanical adulterants most often used for powdered saffron adulteration, but the study also aimed at finding unique markers which could be used for a simple and fast routine analysis by UHPLC-MS/MS.

## Materials and methods

### Chemicals

Deionized water was obtained from a Milli-Q purification system supplied by Millipore (USA); ethanol (96 %, *v/v*) was obtained from Lach-Ner (Czech Republic), HPLC grade methanol from Honeywell (Germany), formic acid from VWR Chemicals (Great Britain), ammonium formate from Merck (Germany), and APCI Positive and Negative Calibration Solutions for the SCIEX X500 System from SCIEX (Canada).

### Samples

In this project, stigmas of 23 different samples of saffron (*Crocus sativus* L.) and 7 samples of saffron powder were analyzed. Regarding geographic origin, 12 samples of saffron stigmas were from Iran, 4 from Spain, and 1 from Greece. The declaration of the geographic origin of other samples was unknown. In addition, potential adulterants were analyzed. The following samples of botanicals were available: 4 safflower petals (*Carthamus tinctorius* L.), 9 calendula petals (*Calendula officinalis* L.), 6 turmeric rhizomes (*Curcuma longa* L.), 3 achiote seeds (*Bixa orellana* L.), 6 dried fruit of red pepper (*Capsicum spp*.), 3 mountain arnica petals (*Arnica montana* L.), 3 bulbs of red beet (*Bea vulgaris* L.), 2 pomegranate petals (*Punica granatum* L.), and 3 pomegranate seeds. The samples of saffron stigmas and potential adulterants were obtained at the Czech market by credible traders with spices.

### Sample preparation

The extraction procedure was based on an earlier published study [31]. Briefly, 50 mg of thoroughly homogenized samples was weighed into 15-mL polypropylene tubes, and 5 mL of the ethanol/water mixture, (7/3, *v/v*) was added. The content of the tube was sonicated for 20 min, and then the sample was centrifuged (5 min, 5 °C, 6000 rpm). The aliquot of the supernatant was transferred into a 2-mL vial and analyzed. All samples were prepared in duplicate.

In the next phase, mixtures of saffron and individual potential adulterants (*w/w* ratios 90/10, 95/5, 97/3, and 99/1) were prepared for analysis. These mixtures were prepared separately for each saffron-adulterant pair. For the preparation of these mixtures, all samples of saffron’s stigmas and separately individual adulterants that were available in this study were mixed. This step was taken to cover possible sample variability. The total weight of the prepared mixture was 100 mg, so twice the volume of solvent was added, i.e., 10 mL of ethanol/water (7/3, *v/v*). Subsequent extraction was performed under the conditions described above. All mixtures of saffron and adulterants were prepared in triplicate.

### Instrumental conditions

The chromatographic separation of sample extracts was performed using a Dionex UltiMate 3000 RS UHPLC system (Thermo Fisher Scientific, USA) with a reversed-phase Acquity UPLC HSS T3 column (2.1 × 100 mm, 1.8 µm particle size, Waters, USA) held at 45 °C. The mobile phase consisted of water (A) and methanol (B), and both containing 5 mM ammonium formate and 0.1 % (*v/v*) formic acid. The mobile phase flow rate was 0.4 mL/min, and the gradient was as follows, 0–1 min 95% (A), 1–8 min 95–0% (A), 8–13 min 0% (A), 13–13.1 min 0–95% (A), and 13.1–15 min 95% (A); the sample injection volume was 2 µL.

Mass spectrometric detection was performed using a TripleTOF 6600 instrument (SCIEX, Canada). The ion source was a Duo Spray with a separated ESI ion source and atmospheric pressure chemical ionization (APCI). ESI was used for the measurement of extracts of the sample, and APCI was used for the exact mass calibration of the instrument. The parameters of the ESI ion source were as follows: capillary voltage, + 5000 V in ESI+ and − 4500 V in ESI−; collision energy, 35 ± 15 V in ESI+ and − 35 ± 15 V in ESI−; declustering potential, 80 V in ESI+ and − 80 V in ESI−; desolvation temperature, 480 °C; curtain gas, 35 psi; drying gas pressure, 55 psi; and nebulizing gas pressure, 55 psi. TOF MS method and information-dependent acquisition (IDA) method were employed to record full MS and MS/MS spectra at the same time. The *m/z* range was between 100 and 1200 Da for MS and between 50 and 1200 Da for MS/MS. An automatic *m/z* calibration was performed every 8 samples using an APCI positive or negative calibration solution for the AB SCIEX TripleTOF^TM^ Systems (SCIEX, Canada).

### Quality control

To check the absence of carry-over effects and to control the stability of recorded fingerprints, blank and quality control (QC) matrix samples were analyzed within UHPLC-HRMS/MS sequences. The in-batch sequence of tested samples was random to avoid any possible time-dependent changes during analysis, which could result in false clustering. To control the overall performance of the instrumental system, QC samples were inserted into the sequence, always after a set of ten tested samples, and analyzed under the same conditions. The QC sample was prepared as a mixture of all analyzed samples. The good instrument performance was documented by a tight clustering of these QC samples (i.e., similarity of their fingerprints) in the principal component analysis (PCA) score plot. The instrument control was carried out with the Analyst TF 1.7.1 software (SCIEX, Canada) and PeakView 2.2 software (SCIEX, Canada).

### Data processing and statistical analysis

The software MarkerView 1.2.1 (SCIEX, Canada) was used for pre-processing raw data generated by the UHPLC-HRMS/MS instrument, separately for ESI+ and ESI−. For the detection of peaks in the range of retention time 0.5–10.5 min, the following parameters were applied: retention time tolerance 0.1 min; mass tolerance 0.01 Da; and data deisotoping. Also, the total area sum normalization (areas of all features in one sample were totaled, and the area of each feature in this sample was divided by this sum; such processing was performed separately for all samples) of the data set was performed, and before the principal component analysis (PCA), the Pareto scaling and logarithmic transformation were applied to all datasets. Different multivariate analysis techniques, such as PCA and partial least squared discriminant analysis (PLS-DA) for the identification of the most significant features, were employed by SIMCA software v. 13.0.3 (Umetrics, Sweden). The model performance was expressed by the predictive ability parameter (*Q*^2^) and by the parameter known as “a goodness of fit” (R^2^(Y)), which was calculated by 7-fold internal cross-validation (6/7 of analyzed samples were used as a training data set and remaining 1/7 of analyzed samples served as a test set; this process was repeated 7 times, so each sample was part of the test set; the distribution into the training and test set was done randomly, but the individual groups were represented equally).

### Selection of the marker ions (markers)

For ranking the marker ions according to their importance, the VIP (variable importance in the projection) plots were used. To illustrate the role of these ions as markers, trend plots were constructed. Another filtration was performed by MetaboAnalyst open-source software v 5.0 (https://www.metaboanalyst.ca), which was used for receiver operating characteristics (ROC). The area under the ROC curve (AUC), *t*-test false discovery rate (FDR) adjusted *p*-value, and fold change (FC) parameters were determined for the created binary models of saffron stigmas and potential plant adulterants. The most important variables with VIP scores > 1 and AUC > 0.8 were chosen.

To verify whether the marker is specific for only one or more adulterants, RAW UHPLC-HRMS/MS data and software SCIEX OS 1.5 (SCIEX, Canada) were used. This software was also used to determine the lowest detectable amount of potential adulterant in a mixture with saffron when using respective marker ions. The detectability of individual marker ions in extracted ion chromatograms was carefully controlled; the lowest detectable amount of respective adulterant in a mixture of saffron stigmas and individual adulterants corresponded to signal-to-noise ratio of 10:1.

### Identification of marker ions (markers)

The tentative identification of markers was based on the calculation of the elemental formula (exact mass and mass error for respective *m/z* values in MS^1^ were considered) and isotopic pattern in MS spectra. To confirm the suggested identification of markers ions, their product ions were investigated in MS/MS spectra. For compound identification, online databases such as ChemSpider (http://www.chemspider.com/), mzCloud (https://www.mzcloud.org/home), PubChem (https://pubchem.ncbi.nlm.nih.gov/), or METLIN (https://metlin.scripps.edu/index.php) were employed.

### Confidence level of identified markers

A confidence level was determined for each marker according to the information in the previous studies [32, 33]. This system divides the markers into levels based on information about their structure, whether the exact 3D or 2D structure, probable structure, or formula are known. In this work, the markers were divided into five categories: levels 0, 1, 2, 3, and 4 [32].

For level 0, it is important to know unambiguous 3D structure including full stereochemistry. If the 2D structure is known or the analytical standard of a marker is available, this is level 1. Level 2 includes substances for which a probable structure or class is known, so it was possible to match the MS/MS spectrum with literature or online databases. If the structure is known, but more isomers are possible, this compound belongs to level 3. If the marker is detected in the sample, but it is not possible to determine its structure, it belongs to level 4 [32].

### Creation of an internal database of markers

The internal database of markers was created using LibraryView 1.0.3. software (SCIEX, Canada) and was subsequently imported into the SCIEX OS 1.5 software (SCIEX, Canada). The formula or exact measured mass, retention time, and MS/MS spectra of all markers are saved in this database. This software was also used to evaluate unknown samples in which markers from the database were searched.

### Evaluation of unknown samples

To control the authenticity of unknown saffron samples in terms of their dilution by some plant adulterants, a targeted screening of 82 markers shown in Table S1 – Markers of saffron and potential adulterants and their characteristics in Supplementary materials was performed. The internal database of these markers was included in the SCIEX OS 1.5 software. This software enabled the comparison of the retention time, exact mass, isotopic pattern in the MS spectrum, and fragments in the MS/MS spectrum of the marker in the measured sample and in the library. The analyst will then check these results. To confirm that the analyzed sample is authentic saffron, all saffron markers from the internal library should be detected, and their relative abundance should not be significantly different from the values in Table S2**–** Relative abundance of saffron markers in samples of stigmas and saffron powder in Supplementary materials. A sample would be considered adulterated supposing more than 80% of the markers of one adulterant were detected. The limit of 80% is based on the Pareto principle.

## Results and discussion

### Method development

**Fig. 1:**
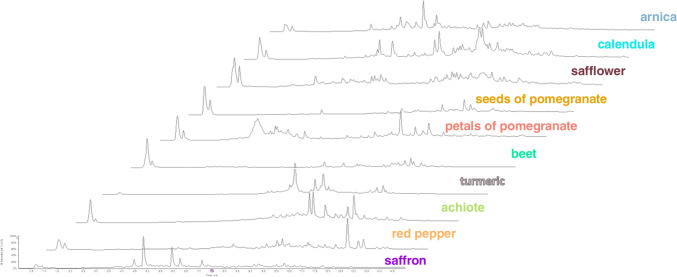
UHPLC-(ESI+)-HRMS fingerprints of saffron and its potential adulterants

**Fig. 2:**
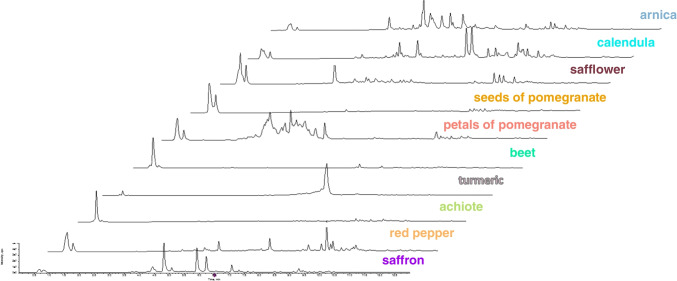
UHPLC-(ESI-)-HRMS fingerprints of saffron and its potential adulterants

Untargeted analysis in combination with chemometrics was employed for the search of specific markers enabling the detection of saffron dilution by particular plant adulterants. UHPLC-HRMS/MS-based approach, rather similar to that earlier developed for the identification of saffron geographic origin [31], was used for the analysis of metabolites extracted by aqueous ethanol from both saffron samples and all possible plant adulterants. In the first phase, the chromatographic separation of sample extract components and mass spectrometric detection of contained metabolites were optimized. The final conditions are described in the “Instrumental conditions” section. Figures 1 and 2 document pronounced differences in UHPLC-HRMS fingerprints obtained from the analysis of saffron, arnica, calendula, safflower, turmeric, achiote, red pepper, seeds, and petals of pomegranate and red beet both in ESI+ and ESI− mode.

**Fig. 3:**
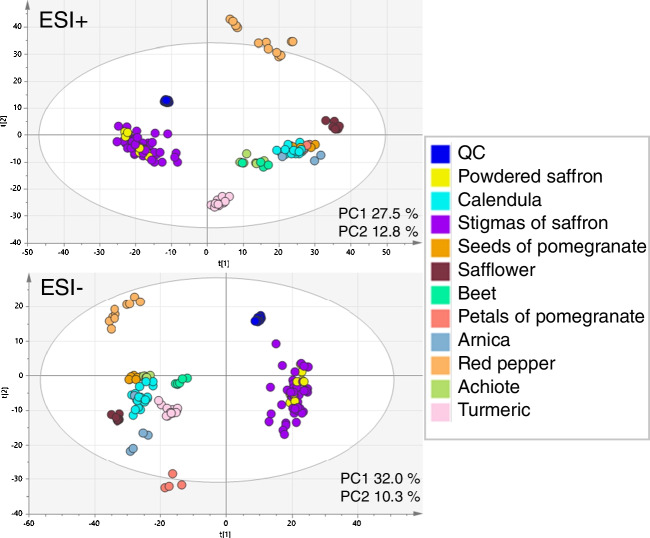
PCA score plots showing a classification of saffron and its potential adulterants; ESI+ and ESI−

After peak detection and their alignment (parameters listed in the “Data processing and statistical analysis” section) within the sample set, using MarkerView software, a list of 5736 molecular features for the ESI+ and 5511 molecular features for the ESI− was obtained (sum for all investigated plant matrices). These final datasets were exported into SIMCA software for statistical analysis. In the first step, the data were processed by an unsupervised tool, PCA, to give a first view of the data structure. The PCA score plot (Fig. 3) showed clustering according to the plant species, reflecting natural variability. In both ionization modes, saffron samples, although rather scattered, were very well separated from other plant species. Incomplete mutual separation of potential adulterants does not pose a problem, as the main aim of this study, reliable distinguishing of saffron, was achieved. Worth to mention, that mainly saffron powder is a target of adulteration [5]. A close clustering with saffron stigmas confirmed not only the authenticity of tested samples but also the minimum impact of the disintegration process on the recorded fingerprint. From the previous study, it is known that the way of postharvest processing (drying) is responsible for some (minor) metabolome changes [31].

In the follow-up phase, only stigmas of saffron were included in the analysis of the principal component analysis together with the individual tested adulterants. Further, partial least squares discriminant analysis was performed for pairs of saffron and other individual potential adulterants. In Table 2, created binary models are characterized. As shown here, the calculated “predictive ability” parameter *Q*^2^ and “a goodness of fit” parameter *R*^2^(*Y*) were very high, which indicates excellent models.

**Table 2:** "Predictive ability parameters” (*Q*^2^) and “goodness of fit” parameters (*R*^2^(*Y*)) of the created binary PLS-DA models for the classification of saffron stigmas and potential plant adulterants

Potential adulterant of saffron stigmas	ESI+		ESI−	
	*R*^2^(*Y*)	*Q*^2^	*R*^2^(*Y*)	*Q*^2^
Safflower	0.999	0.993	0.999	0.996
Calendula	0.999	0.997	0.998	0.996
Arnica	0.998	0.988	0.998	0.990
Turmeric	0.996	0.992	0.996	0.992
Achiote	0.995	0.985	0.996	0.993
Petals of pomegranate	0.998	0.990	0.997	0.992
Seeds of pomegranate	0.995	0.989	0.996	0.991
Beet	0.992	0.986	0.995	0.993
Red pepper	0.997	0.992	0.998	0.996

The models created in this way (stigmas of saffron, an adulterant) were also used to the selection of the characteristic markers of individual potential adulterants and saffron. Variable importance plots were constructed. For this purpose, only ions with VIP score > 1 and simultaneously with AUC > 0.8 were investigated as potential “markers.”

**Fig. 4:**
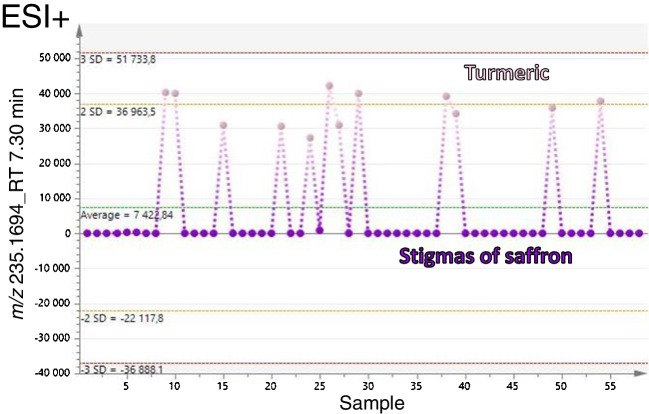
Trend plot of turmeric marker (*m/z* 235.1694; retention time 7.30 min; predicted formula C_15_H_22_O_2_) in saffron stigmas and turmeric samples; ESI+

For the demonstration of marker ion specificity, a trend plot for each marker was used. An example is shown in Fig. 4. As documented in this Fig. 4, metabolite corresponding to the ion *m/z* 235.1694 (retention time: 7.30 min; predicted formula: C_15_H_22_O_2_) was not present in any of the saffron samples. Due to the unavailability of fragmentation spectra in the used databases, it is not possible to obtain unbiased identification of the marker. Based on the exact mass and isotopic pattern in MS spectra, it is probably one of the following substances: dihydrocostunolide, isocurcumenol, or curcumenol.

In the next phase, UHPLC-HRMS/MS data were assessed using SCIEX OS software to check the specificity of saffron markers, i.e., whether they cannot be detected also in other adulterants. As far as was the case, they were removed from the list of markers. A similar approach was employed to control whether compounds identified as markers in adulterants are not present in saffron. On the other hand, their presence in more than one plant species was not the reason for excluding them from the markers list.

**Fig. 5:**
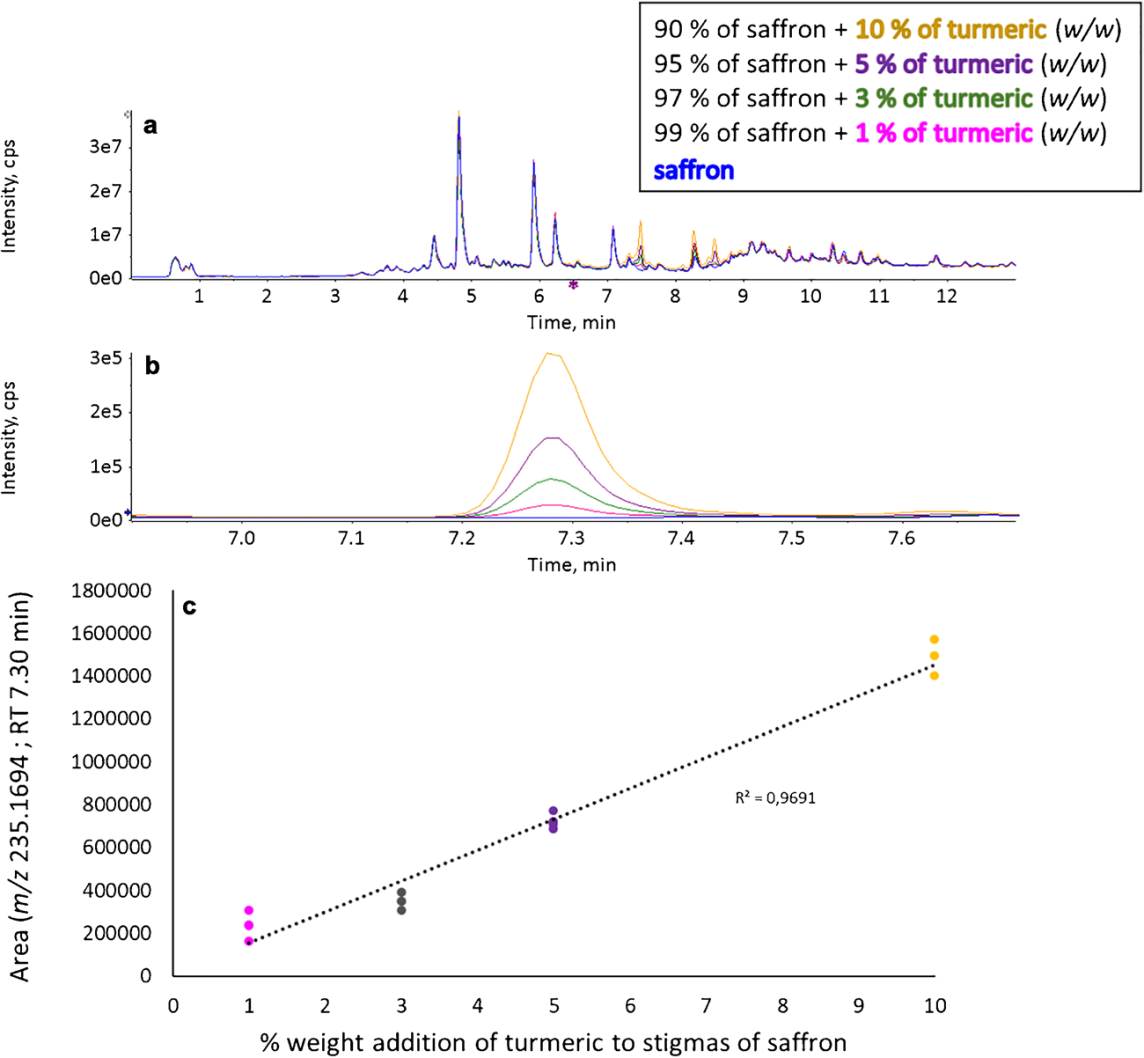
**a **UHPLC-(ESI+)-HRMS fingerprints of saffron stigmas and different mixtures of saffron and turmeric; **b** extracted ion chromatogram of turmeric marker (*m/z* 235.1694; retention time 7.30 min; predicted formula C_15_H_22_O_2_) in extracts of saffron stigmas and different mixtures of saffron and turmeric; **c** linear dependence of the peak area of the turmeric marker (*m/z* 235.1694; retention time 7.30 min; predicted formula C_15_H_22_O_2_) on the amount of turmeric in mixtures with saffron: ESI+

The verification of the suitability of adulterant markers for fraud detection continued by testing their signal increase by increasing the amount of respective plant in a mixture with saffron. An example of a relevant turmeric marker is shown in Fig. 5. The lowest detectable amount of potential adulterant in a mixture with saffron was determined based on the signal-to-noise ratio (detailed procedure in the “Selection of the marker ions” section), and the results are shown in Table S1 – Markers of saffron and potential adulterants and their characteristics in the Supplementary materials. Regarding the lowest detectable amount of potential adulterant, all the investigated plants were even at a 1% (*w/w*) addition level detectable in at least three marker ions with the signal-to-noise ratio of at least 10:1. It is worth to mention that comparable results were achieved only in studies [15, 19] using molecular genetic methods (Table 1); nevertheless, none of them involved such many potential saffron adulterants as this study.

As soon as marker ions were selected, at least tentative identification was suggested. Some substances were identified as known secondary metabolites — like safranal, crocetin, hydroxysafflower yellow A, and capsaicin. The highest mass error between the predicted and measured values was −4.8 ppm. All marker information, including mode of ionization, retention time, exact mass, MS/MS spectrum, predicted formula, adduct, mass error, tentative identification, confidence level, the lowest detectable amount in mixture with saffron using this marker, and statistical parameter like AUC,* p*-value, FC a VIP score, are summarized in Table S1 – Markers of saffron and potential adulterants and their characteristics in Supplementary materials.

For target screening, the internal spectral database was created using the LibraryView software, which was then inserted into the SCIEX OS 1.5 software. The database currently contains 82 markers, 53 detectable in ESI+, and 29 in ESI−. The formula or exact measured mass, retention time, and the MS/MS spectra of markers were saved in the internal database, and these information were used for evaluating the sample.

The knowledge of such markers enables significant simplification of saffron authentication, as the processing of data obtained by target screening is fairly less time demanding; moreover, a triple quadrupole mass analyzer can be used instead of a high-resolution one, which is needed for metabolic fingerprinting.

### Application of the developed method (target screening of markers) to powdered saffron samples from the Czech market

This library of markers was tested to evaluate 7 samples of saffron powder from the Czech market. These samples were previously analyzed together with the set of samples of saffron stigmas, as shown in the PCA score plot (Fig. 3). Target screening of markers confirmed that these samples were not adulterated. All saffron markers were detected in these samples, and any adulterant markers were not detected.

**Fig. 6:**
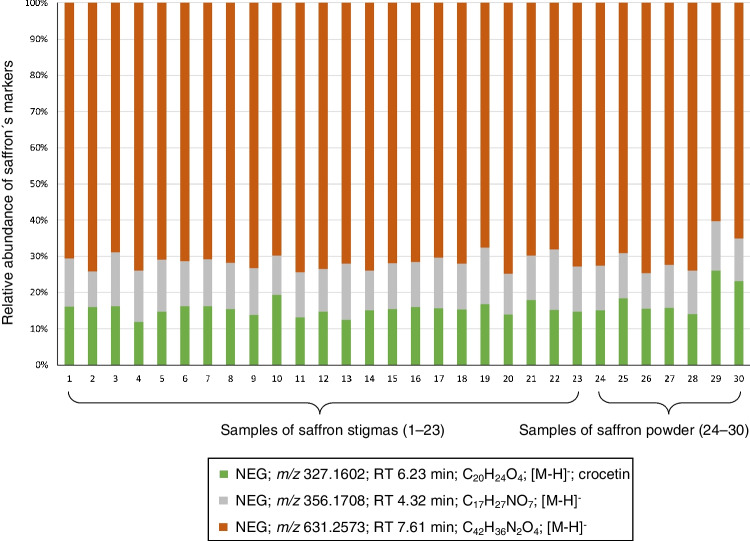
Relative abundance of saffron markers in samples of stigmas and saffron powder; ESI+

**Fig. 7:**
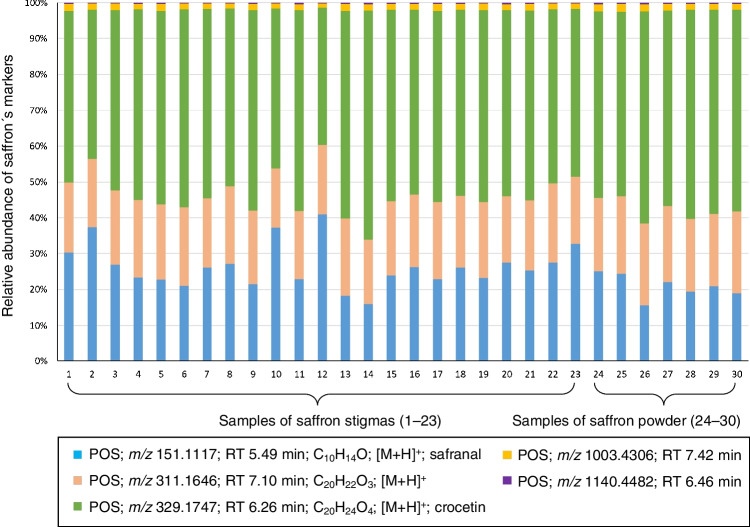
Relative abundance of saffron markers in samples of stigmas and saffron powder; ESI−

The relative abundance of saffron markers in samples of stigmas and powdered saffron was also compared. This was determined by calculating the sum of the areas of all saffron markers in one sample (separately for positive and negative electrospray ionization modes), and each area was divided by this sum. The value was converted to percentages and shown in Figs. 6 and 7 and Table S2** –** Relative abundance of saffron markers in samples of stigmas and saffron powder in Supplementary materials. As can be seen, the results in both saffron stigmas and powder were similar.

## Conclusions

Within this study, a simple and fast method was developed, which enabled the detection of powdered saffron dilution/substitution by any of the eight most often used botanical adulterants represented by petals of safflower (*Carthamus tinctorius L.*), petals of calendula (*Calendula officinalis* L.), rhizomes of turmeric (*Curcuma longa* L.), seeds of achiote (*Bixa orellana* L.), dried fruits of red pepper (*Capsicum* spp.), petals of mountain arnica (*Arnica montana* L.), bulbs of red beet (*Beta vulgaris* L.), and petals and seeds of pomegranate (*Punica granatum* L.). Metabolomic fingerprinting (non-target screening) of aqueous ethanolic saffron/plant extracts obtained by UHPLC-HRMS/MS followed by multivariate statistical processing of generated data sets resulted in the selection of 82 unique marker ion characteristic for tested plant species. Targeted screening of these markers can be used for unbiased authenticity evaluation of samples labeled as “powdered saffron.” Addition as low as 1 % (*w/w*) of botanical adulterant to saffron powder is possible to detect, which is “sensitivity” comparable to that achievable by fairly more demanding methods based on molecular biology. It is worth to notice that while searching for markers requires the use of an instrumental platform with a high-resolution mass analyzer, the knowledge of concrete markers could allow a simple targeted analysis by widely available instruments equipped with a common triple quadrupole mass analyzer.

## Supplementary Information

Below is the link to the electronic supplementary material.

Supplementary file1 (XLSX 34 KB)

Supplementary file2 (DOCX 20 KB)
