# Supplementary material for: Detection of botanical adulterants in saffron powder
Source: Anal Bioanal Chem. 2023 Aug 17;415(23):5723–34. doi: 10.1007/s00216-023-04853-x (PMC10474180; doi:10.1007/s00216-023-04853-x)
Supplement: Supplementary file 2 — Supplementary file2 (DOCX 20 KB) [file 216_2023_4853_MOESM2_ESM.docx]

**Table S2 –** Relative abundance of saffron markers in samples of stigmas and saffron powder

| **Saffron markers** | **Relative abundance of saffron markers in saffron stigmas (%)** | **Relative abundance of saffron markers in saffron powder (%)** |
| --- | --- | --- |
| POS; *m/z* 151.1117; RT 5.49 min; C_10_H_14_O; [M+H]^+^; safranal | 16 – 41 | 16 – 25 |
| POS; *m/z* 311.1646; RT 7.10 min; C_20_H_22_O_3_; [M+H]^+^ | 17 – 22 | 20 – 23 |
| POS; *m/z* 329.1747; RT 6.26 min; C_20_H_24_O_4_; [M+H]^+^; crocetin | 38 – 64 | 52 – 59 |
| POS; *m/z* 1003.4306; RT 7.42 min | 1.2 – 2.0 | 1.6 – 2.2 |
| POS; *m/z* 1140.4482; RT 6.46 min | 0.18 – 0.48 | 0.27 – 0.48 |
| NEG; *m/z* 327.1602; RT 6.23 min; C_20_H_24_O_4_; [M-H]^-^; crocetin | 12 – 19 | 14 – 26 |
| NEG; *m/z* 356.1708; RT 4.32 min; C_17_H_27_NO_7_; [M-H]^-^ | 10 – 17 | 10 – 14 |
| NEG; *m/z* 631.2573; RT 7.61 min; C_42_H_36_N_2_O_4_; [M-H]^-^ | 68 – 75 | 60 – 75 |
